# The neurocognitive correlates of DTI indicators of white matter disorganization in pediatric moderate-to-severe traumatic brain injury

**DOI:** 10.3389/fnhum.2024.1470710

**Published:** 2024-10-31

**Authors:** Daniel A. Ignacio, Talin Babikian, Emily L. Dennis, Kevin C. Bickart, Meeryo Choe, Aliyah R. Snyder, Anne Brown, Christopher C. Giza, Robert F. Asarnow

**Affiliations:** ^1^Steve Tisch Brain SPORT Program, University of California, Los Angeles, Los Angeles, CA, United States; ^2^Department of Psychiatry and Biobehavioral Sciences, Semel Institute for Neuroscience and Human Behavior, University of California, Los Angeles, Los Angeles, CA, United States; ^3^Department of Neurology, School of Medicine, The University of Utah School of Medicine, Salt Lake City, UT, United States; ^4^Department of Neurology, David Geffen School of Medicine, University of California, Los Angeles, Los Angeles, CA, United States; ^5^Division of Pediatric Neurology, UCLA Mattel Children’s Hospital, Los Angeles, Los Angeles, CA, United States; ^6^Department of Clinical and Health Psychology, College of Public Health and Health Professions, University of Florida, Gainesville, FL, United States

**Keywords:** white matter organization, frontoparietal network, precentral cortex, postcentral cortex, moderate/severe pediatric TBI, diffusion tensor imaging, interhemispheric transfer time, working memory

## Abstract

**Introduction:**

Neuroimaging has expanded our understanding of pediatric brain disorders in which white matter organization and connectivity are crucial to functioning. Paralleling the known pathobiology of many neurodevelopmental disorders, traumatic brain injury (TBI) in childhood can alter trajectories of brain development. Specifically, diffusion tensor imaging (DTI) studies in TBI have demonstrated white matter (WM) abnormalities that suggest microstructural disruptions that may underlie atypical neurodevelopment. The neurocognitive correlates of these previous findings will be explored in this study.

**Methods:**

Indicators of WM organization were collected in 44 pediatric patients with moderate/severe TBI and 76 controls over two post-injury time points: T1 (8–20 weeks) and T2 (54–96 weeks). Our previous work identified two TBI subgroups based on information processing differences: one with slower interhemispheric transfer times (IHTT) of visual information than controls and another with comparable IHTT. We extend this prior work by evaluating neurocognitive trajectories associated with divergent WM structure post-injury in slow and normal IHTT TBI subgroups.

**Results:**

At T1, both TBI subgroups performed significantly worse than controls on a norm-referenced working memory index (WMI), but only the Normal IHTT TBI subgroup significantly improved over the 12-month follow-up period (*p* = 0.014) to match controls (*p* = 0.119). In contrast, the Slow IHTT TBI subgroup did not show any recovery in working memory performance over time and performed more poorly than the control group (*p* < 0.001) at T2. Improvement in one of the two WMI subtests was associated with DTI indicators of WM disorganization in CC tracts to the precentral, postcentral, frontal, and parietal cortices. IHTT and WM mean diffusivity predicted 79% of the variance in cognitive recovery from T1 to T2 when also accounting for other known predictors of TBI recovery.

**Discussion:**

In the year following TBI, some pediatric patients experienced persisting working memory disturbance while others exhibited recovery; stratification was based on an event-related potential marker. More or less improvement in neurocognition was also associated with the degree of WM disorganization. IHTT, measured post-acutely after TBI, and progression of WM disorganization over time predicted neurocognitive trajectories at the chronic timeframe - potentially representing a prognostic biomarker.

## Introduction

The development of typical brain anatomy and physiology is an intricate process of generating diverse cell types and assembling them into highly organized functional circuits that underlie cognitive and emotional functioning. Whereas neurogenesis and neuronal migration occur in the first year of human life, processes such as axonogenesis, synaptogenesis, and myelination of white matter (WM) continue through early adulthood (Khodosevich and Sellgren, 2022) and changes in WM structure via activity-dependent myelination continue throughout the lifespan (de Faria et al., 2019; Sampaio-Baptista and Johansen-Berg, 2017). Using tract-based spatial analysis, Li et al. (2022) revealed that working memory functioning relies on WM organization, with the strongest effect in the corpus callosum (CC), which was also suggested to contribute to age-related differences in working memory. Myelinated WM tracts, particularly in the CC, are vulnerable to axonal shearing and traumatic axonal injury, or TAI (Molteni et al., 2019). Despite typical early neurodevelopment, significant disruptions to the developmental trajectory can occur throughout a child’s life. One such disruption, and the focus of this manuscript, is pediatric traumatic brain injury (TBI), which occurs during active neurodevelopment and can significantly alter neurotypical trajectories (Roberts and Mathias, 2014). TBI is associated with WM disorganization, partly due to TAI, that impacts efficient brain function and WM maturation (Dennis et al., 2015).

Working memory, which is a common concern after TBI (Ewing-Cobbs et al., 2006; Gorman et al., 2011), is a capacity that develops throughout childhood through WM microstructure maturation (Krogsrud et al., 2018) and is foundational for a broader range of high order cognitive abilities such as reasoning, comprehension, problem-solving, and academic achievement (Sesma et al., 2008; Conklin et al., 2008). Functional difficulties for children across settings are precipitated by behavioral or learning problems potentially exacerbated by deficits in working memory, although the understanding of specific relationships between WM microstructure and different aspects of working memory is currently limited (Krogsrud et al., 2018).

Advances in Diffusion Tensor Imaging (DTI) tractography have enabled the study of anatomically defined regions and behavioral parameters of functionality like neurocognition (Sasson et al., 2013). For example, a developmental study of 296 children from the Norwegian Mother and Child Cohort Study revealed moderate support for associations between DTI indicators, such as mean diffusivity (MD) and fractional anisotropy (FA), within specific WM tracts (e.g., genu of CC) and visuospatial working memory, but not verbal working memory (Krogsrud et al., 2018). According to Sousa et al. (2018), the absence or damage to myelin in WM tracts is not a key factor for anisotropic diffusion to exist (Beaulieu, 2002) and does not appear to be a condition that changes FA; however, myelin pathology may be important for changes in MD since water displacement is greater in the perpendicular direction, rather than parallel, to axon bundles. Furthermore, increased MD values were proposed to be associated with myelin lesions and cognitive impairment in patients with demyelinating WM disorders (Preziosa et al., 2017). Alternative to the findings above, both verbal and non-verbal memory were associated with FA and other diffusivity metrics in the WM tract that connect the frontal, parietal, temporal, and occipital lobes in healthy adults aged 25 to 82 years-old (Sasson et al., 2013). Indeed, the available developmental studies have been inconclusive in regard to regional specificity with WM tract microstructure and working memory (Krogsrud et al., 2018; Thomason et al., 2009; Vestergaard et al., 2011).

As a complement to WM microstructural organization, a functional CC measure of interhemispheric transfer time (IHTT) has been suggested to mediate associations between microstructure and outcomes in patients following pediatric TBI (Ellis et al., 2016; Moran et al., 2016). IHTT refers to the latency of information presented to one visual field that is then registered in the contralateral hemisphere, which can be more directly measured using electroencephalography (EEG) scalp recordings of visual event-related potentials (Ellis et al., 2016). In a pediatric sample following moderate-to-severe TBI, a bimodal distribution of children was revealed, one with IHTT comparable to controls and the other with significantly slower IHTT. These IHTT subgroups were further characterized to have divergent trajectories of CC microstructural recovery over one-year post-injury (Dennis et al., 2017). While the pediatric neuroimaging literature has revealed persisting structural differences as a result of TBI, no clear connection between functional and cognitive outcomes has been presented (Guerriero and Schlaggar, 2017).

### The present study

We evaluated the longitudinal trend of neurocognitive functioning as measured by verbal working memory, which is affected by WM organization and critically important for learning. We examined whether longitudinal changes in verbal working memory paralleled that of our previous finding in DTI microstructure (Dennis et al., 2017). To evaluate the evolution of WM diffusion and working memory disturbances, participants were first assessed in the post-acute stage (< 6 months) and then their measurements were correlated with long-term outcomes (> 6 months), as per empirical convention (Sidaros et al., 2008). After a TBI in childhood, recovery has been proposed to be most pronounced after the first- and second years following injury (Yeates et al., 2002). In a 5-year longitudinal prospective cohort, Dinkel et al. (2014) also revealed microstructural changes in the CC between baseline and up to two years following injury with stability between two and five years; moreover, diffusivity indicators in the genu and body of the CC were associated with neurocognitive symptoms (e.g., amnesia, dyspraxia, aphasia). Furthermore, based on our previous work (Ellis et al., 2016; Moran et al., 2016), we evaluated whether IHTT predicted cognitive performance over time and evaluated the correlation between cognitive scores and microstructural indices of WM damage in verbal working memory neural networks, such as the frontoparietal network (Chai et al., 2018; Østby et al., 2011). This helped address the question of how well IHTT predicts chronic outcomes. These results provided insight into the neural mechanisms underlying verbal working memory and highlight the importance of WM organization in cognitive performance.

## Materials and methods

### Participants

There were 125 participants evaluated at Timepoint 1 (T1) and 90 participants evaluated at Timepoint (T2). For sample demographics, refer to Table 1. Of the seventy-five participants who completed both visits, 30 were patients with TBI who were initially evaluated 8-20 weeks post-injury (T1) and again 54-96 weeks post-injury (T2). Patients were recruited from pediatric intensive care units from Los Angeles County (UCLA Medical Center and Harbor-UCLA Medical Center) and a Los Angeles based-rehabilitation hospital. Patients were included in this study if they met the following criteria: (1) moderate to severe non-penetrating TBI (intake or post-resuscitation GCS score between 3 and 12, or higher GCS score with confirmed abnormalities on clinical imaging); (2) 8–18 years of age at injury; (3) normal visual acuity or vision corrected with contact lenses/eyeglasses; and (4) English skills sufficient to understand instructions and participate in the neurocognitive measures. Patients with a pre-trauma history of neurological, developmental, or psychiatric disorders (including prior head injury) or MR-incompatible metal implants were excluded. Typically developing controls were recruited from the community through flyers, magazines, and school postings using the same inclusion and exclusion criteria, where appropriate.

**TABLE 1:** Demographic information for the TBI sample and TD controls over time.

	TBI		
	Slow IHTT	Normal IHTT	TD Controls
T1 (*n* = 120); T2 (*n* = 90)	19	25	76
Sex (M/F)	12/7	20/5	41/35
Age (*SD*), in years: T1	14.37 (2.15)	14.75 (3.42)	15.00 (2.93)
Age (*SD*), in years: T2	15.30 (2.63)	16.21 (3.14)	15.83 (2.88)
Years of Parent Education	13.05 (3.61)*	13.72 (3.53)	15.28 (3.37)*
GCS score on hospital arrival (*SD*)	8.84 (4.11)	8.63 (4.44)	–
Weeks (*SD*) Since Injury: T1	12.10 (4.74)	13.46 (5.11)	–
Weeks (*SD*) Since Injury: T2	65.87 (8.28)	67.67 (7.68)	–
	**TBI**		
	**Slow IHTT**	**Normal IHTT**	**TD Controls**
Both T1/T2 (*N* = 75)	16	14	45
Sex (M/F)	11/5	10/4	27/18
Age (*SD*), in years: T1	14.70 (1.99)	15.06 (3.19)	15.11 (2.59)
Age (*SD*), in years: T2	15.73 (2.00)	16.12 (3.15)	16.27 (2.63)
Years of Parent Education	12.94 (3.87)*	14.36 (4.09)	16.07 (3.05)*
GCS score on hospital arrival (*SD*)	9.13 (4.06)	8.77 (4.49)	–
Weeks (*SD*) Since Injury: T1	12.13 (4.69)	12.67 (5.67)	–
Weeks (*SD*) Since Injury: T2	65.87 (8.28)	68.32 (8.00)	–

*p < 0.05; TBI, traumatic brain injury; TD, typically developing; GCS, Glasgow Coma Scale; IHTT, interhemispheric transfer time; SD, standard deviation; T1, post-acute (*M* = 12.38 weeks, *SD* = 5.08); T2 = chronic (*M* = 67.01 weeks, *SD* = 8.11); Slow IHTT and Normal IHTT were derived based on the bimodal distribution of two subgroups of children with TBI: one group with speeds greater than 1.5 standard deviations lower than typically developing controls and a second group within 1.5 standard deviations of the normative speed (Ellis et al., 2016).

### Measures

#### Neuroimaging and electrophysiological evaluations

**FIGURE 1:**
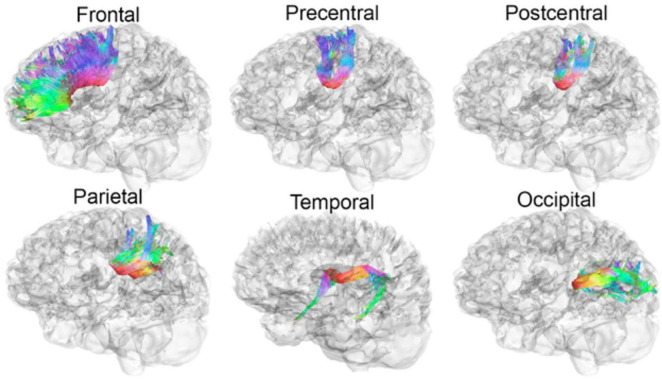
Participants of the current study were scanned on 3T Siemens Trio MRI scanners with whole brain anatomical and 66-gradient DWI (2mm^3^ voxel size, b = 1000 s/mm^2^ see full description Dennis et al., 2015). Automated multi-atlas tract extraction was applied on DWI to measure MD in six CC tracts, as detailed in a prior study (Dennis et al., 2017). Evaluated WM tracts from the CC to the frontal, precentral, postcentral, parietal, temporal, and occipital cortices are illustrated below. MRI, magnetic resonance imaging; DWI, diffused weighted imaging; MD, mean diffusivity; CC, corpus callosum.

Mean diffusivity was computed from DTI within prominent WM tracts through the CC to assess microstructural organization. Participants were scanned on 3T Siemens Trio MRI scanners with whole brain anatomical and 66-gradient DWI (2mm^3^ voxel size, b = 1000 s/mm^2^; see full description Dennis et al., 2015). Automated multi-atlas tract extraction was applied on DWI to measure MD in six CC tracts (Figure 1), as detailed in a prior study (Dennis et al., 2017). For a functional indicator of WM organization, visual event-related potentials (ERP) were recorded using linked-ears references as opposed to mid-frontal reference points to derive a more valid estimate of IHTT. The IHTT task was performed and two TBI subgroups (i.e., Slow IHTT, Normal IHTT) were identified based on ERP speeds compared to controls as per previously published protocol (see Ellis et al., 2016; Moran et al., 2016 for details). Stimuli were presented to one visual field, and an ERP was measured in the primary visual cortex on the ipsilateral hemisphere then to the homologous site in the contralateral hemisphere; IHTT is difference in latency between the ERP components between the two hemispheres.

#### Neurocognitive evaluations

Participants completed standardized tests to evaluate their verbal working memory abilities via the Wechsler Intelligence Scale for Children, 4^th^ edition, or the Wechsler Adult Intelligence Scale, 3^rd^ edition, depending on their age at evaluation. Working memory was measured with the Working Memory Index (WMI), which is norm-referenced by age. The WMI constituted two tasks, Digit Span (DS) and Letter-Number Sequencing (LNS). DS involved repeating a series of digits spoken by an examiner in two ways with subsequent trials increasing in one additional digit until a maximum capacity is reached: first, the participant is to repeat digits in the same order spoken; second, the participant is to repeat digits in the reverse order from what was spoken. For the LNS task, an examiner would speak a series of both digits and letters with subsequent trials increasing in additional characters and participants must first say the numbers in ascending order and then the letters in alphabetical order.

### Procedure

The overall study was approved by the University of California, Los Angeles (UCLA) institutional review board and the institutional review boards of each facility from which patients were recruited. Participants underwent electrophysiological and neurocognitive evaluations on the same day, or on rare occasions, within at most a 2-week span. The average MRI scan interval was 1.1 years (range 0.4 to 1.9 years).

### Statistical analysis

Data were analyzed using IBM SPSS Statistics 29.0. Analyses reported include independent samples t-tests, Pearson correlations, linear regression, and analysis of covariance (ANCOVA) tests. Age, sex, injury characteristics, and years of parent education were used as covariates. According to the social-cognitive-ecological model (Guerra and Huesmann, 2004), measures of family socioeconomic status, including parent education, were revealed to be strongly predictive of future child outcomes (Dubrow et al., 2009). Furthermore, IQ and education of parents with offspring who had neurodevelopmental concerns were revealed to significantly correlate with their child’s IQ (Meador et al., 2012). These covariates were considered using ANCOVA to remove error variance (“noise”) in our models not related to the independent variable (Miller and Chapman, 2001, p. 42); there was a statistical difference in years of parent education between the control group and the Slow IHTT group (*p* = 0.003), but not within the TBI group (Slow versus Normal IHTT; *p* = 0.264), so this confound was not included in full sample models. There were no statistical differences observed between groups for age (*p* = 0.860), binary sex (*p* = 0.678), or Glasgow Coma Scale (GCS) score at hospital admission (*p* = 0.825).

When considering the randomness of attrition, no notable demographic differences were observed between those who remained in the sample versus those who did not. The available data was subjected to Little’s MCAR test to confirm mechanism of missingness, X^2^ = 40.61, *p* = 0.618; additionally, missingness was sparse. Pairwise deletion was deemed appropriate to manage missing data. Changes in the measurements of interest from T1 to T2 were evaluated by using percent delta (T_2_ – T_1_/ T_1_). After screening scatterplots and boxplots, a single outlier was identified for DS delta (*Z* = +3.15, *p* < 0.01). The scores were reasonable in natural progression and did not represent extreme scores at either T1 or T2, so the participant datum was retained to minimize data loss. Benjamini-Hochberg correction was applied to the presented significance values in the main analyses to control the False Discovery Rate while optimizing power to detect true effects (Glueck et al., 2008).

## Results

**FIGURE 2:**
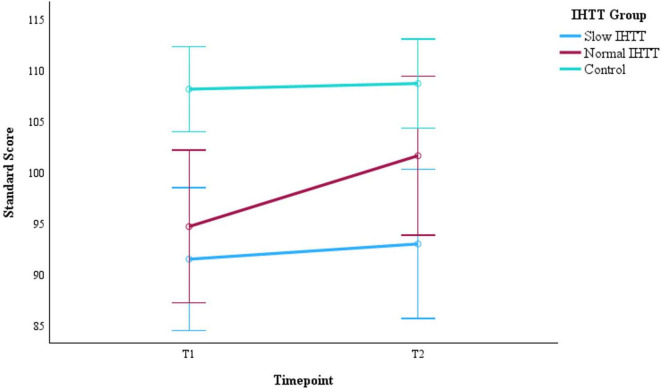
Changes in WMI performance from post-acute (T1) to chronic (T2) for the two subgroups of pediatric patients with TBI stratified by IHTT and the typically developing control group with data at both timepoints (N = 75). WMI is based on standard scores with a mean of 100 and SD of 15. Error bars represent the 95% confidence intervals of the mean. The covariates appearing in the model are evaluated at the following values: Age at T1 = 15.01, Sex = 0.36. WMI, working memory index; T1, 8–20 weeks post injury; T2, 54–96 weeks post injury; TBI, traumatic brain injury; IHTT, interhemispheric transfer time.

For the full sample at T1, using only age and binary sex as covariates, WMI performance was significantly worse for both the Slow IHTT (*p* < 0.001) and Normal IHTT (*p* = 0.015) groups compared to the control group. Over time, only the Normal IHTT group (*p* = 0.014) improved in working memory performance from T1 to T2 to the point where their performance no longer differed from controls (Figure 2). In contrast, the Slow IHTT TBI subgroup did not show any recovery in working memory performance by T2 (*p* = 0.565) and remained significantly lower as compared to the control group (*p* < 0.001) with the Normal IHTT group in the intermediary and non-significant from both control (*p* = 0.119) and Slow IHTT (*p* = 0.110) groups. The control group’s WMI performance stayed consistent from T1 to T2 (*p* = 0.719). WMI is constituted by two tasks, DS and LNS, and is separated below. See Table 2 for overall statistics for WMI, DS, and LNS factored by IHTT.

**TABLE 2:** Overall statistics for the neurocognitive measure of working memory stratified by IHTT groups.

	TBI		
	Slow IHTT	Normal IHTT	TD Controls
**Working Memory Index (WMI)**			
Mean (*SD*)	92.22 (15.33)	98.46 (12.31)	108.36 (12.97)
**Digit Span (DS)**			
Mean (*SD*)	8.66 (2.71)	9.43 (3.30)	11.07 (2.47)
**Letter Number Sequencing (LNS)**			
Mean (*SD*)	8.78 (2.81)	10.14 (1.73)	11.99 (2.39)

IHTT, interhemispheric transfer time; TBI, traumatic brain injury; TD, typically developing; SD, standard deviation. WMI is based on standard scores with a mean of 100 and SD of 15. DS and LNS are based on scaled scores with a mean of 10 and SD of 3.

**FIGURE 3:**
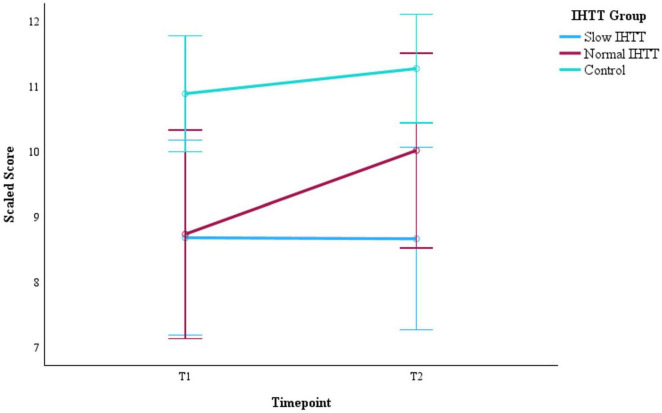
Changes in DS performance from post -acute (T1) to chronic (T2) for the two subgroups of pediatric patients with TBI stratified by IHTT and the typically developing control group with data at both timepoints (*N* = 75). DS is based on scaled scores with a mean of 1O and SD of 3. Error bars represent the 95% confidence intervals of the mean. The covariates appearing in the model are evaluated at the following values: Age at T1 = 15.01, Sex = 0.36. DS = digit span; T1 = 8–20 weeks post injury; T2 = 54–96 weeks post injury; TBI, traumatic brain injury; IHTT, interhemispheric transfer time.

Years of parent education and GCS score at hospital admission were added as additional covariates to evaluate WMI within the TBI group only (Slow IHTT and Normal IHTT subgroups combined), which revealed a significant Group x Time interaction effect, *F*(1, 23) = 5.48, *p* = 0.028, η_*p*_^2^ = 0.192 (Table 3). The Slow IHTT group (*p* = 0.766) did not exhibit the same degree of improvement in WMI performance as the Normal IHTT group (*p* = 0.002) from T1 to T2. There were non-significant main effects of Time (*p* = 0.753) or IHTT group (*p* = 0.446). A similar pattern was observed for DS. There was also a significant Group x Time interaction for DS scores from T1 to T2 within the TBI group, *F*(1, 23) = 6.27, *p* = 0.020, η_*p*_^2^ = 0.214 (Table 4). The Slow IHTT group (*p* = 0.577) at T1 (*M* = 8.99, *SE* = 0.88) did not exhibit the same degree of improvement at T2 (*M* = 8.68, *SE* = 0.71) in DS scaled scores as the Normal IHTT group (*p* = 0.008) from T1 (*M* = 8.48, *SE* = 0.98) to T2 (*M* = 10.24, *SE* = 0.79). A full sample Group x Time ANCOVA (*N* = 75) on DS scaled scores also revealed longitudinal changes in verbal working memory that paralleled that of our previous finding in DTI microstructure (see Dennis et al., 2017); similar to the Slow IHTT group (*n* = 16), the control group (*n* = 45) performed consistently (*p* = 0.252) from T1 (*M* = 10.89, *SE* = 0.45) to T2 (*M* = 11.27, *SE* = 0.42) with only the Normal IHTT group (*n* = 14) improving to the intermediary and non-significant from both control (*p* = 0.148) and Slow IHTT (*p* = 0.191) groups (Figure 3). There were non-significant main effects of Time (*p* = 0.381) and IHTT group (*p* = 0.653). For the LNS task, no significant main (*p* = 0.338) or interaction (*p* = 0.471) effects were revealed.

**TABLE 3:** Descriptive comparisons for WMI performance in TBI subgroups stratified by IHTT at T1 and T2.

Group	Timepoint	$\bar{\text{x}}$ (*SE*)	Δ$\bar{\text{x}}$ (T1-T2)	Δ$\bar{\text{x}}$ (T1)	Δ$\bar{\text{x}}$ (T2)
Slow IHTT (A)	T1	92.90 (4.05)	−0.66	−0.45 (A–B)	−8.22 (A–B)
	T2	93.56 (3.58)			
Normal IHTT (B)	T1	93.35 (4.51)	−8.42 ***	
	T2	101.78 (3.99)		

***p < 0.01; $\bar{\text{x}}$ = mean; SE = standard error; Δ$\bar{\text{x}}$ = mean change. WMI, working memory index; TBI, traumatic brain injury; IHTT, interhemispheric transfer time; T1, post-acute (M = 12.38 weeks, SD = 5.08); T2 = chronic (M = 67.01 weeks, SD = 8.11).

**TABLE 4:** Descriptives for DS scaled scores in TBI subgroups stratified by IHTT at T1 and T2.

Group	Timepoint	$\bar{\text{x}}$ (*SE*)	Δ$\bar{\text{x}}$ (T1-T2)	Δ$\bar{\text{x}}$ (T1)	Δ$\bar{\text{x}}$ (T2)
Slow IHTT (A)	T1	8.99 (0.88)	0.31	0.51 (A–B)	−1.55 (A–B)
	T2	8.68 (0.71)			
Normal IHTT (B)	T1	8.48 (0.98)	−1.76**	
	T2	10.24 (0.79)		

**p < 0.05; $\bar{\text{x}}$, mean; SE, standard error; Δ$\bar{\text{x}}$, mean change. DS, digit span; TBI, traumatic brain injury; IHTT, interhemispheric transfer time; T1, post-acute (M = 12.38 weeks, SD = 5.08); T2 = chronic (M = 67.01 weeks, SD = 8.11).

The differential improvement between groups for DS delta over time was correlated with MD delta in most, but not all, of the evaluated CC tracts. For only the TBI group (Slow IHTT and Normal IHTT subgroups combined; *n* = 30), DS delta was negatively correlated with MD delta over time in CC tracts to the precentral (*r* = −0.468, *p* = 0.043), postcentral (*r* = −0.598, *p* = 0.007), frontal (*r* = −0.577, *p* = 0.010), and parietal cortices (*r* = −0.495, *p* = 0.031), but not the temporal (*p* = 0.243) nor occipital cortices (*p* = 0.073). Average IHTT speeds for patients with TBI at T1 were positively correlated with MD delta in CC tracts to the precentral (*r* = 0.493, *p* = 0.042) and postcentral (*r* = 0.470, *p* = 0.042) cortices, but not the frontal (*p* = 0.298), parietal (*p* = 0.099), temporal (*p* = 0.243), nor occipital (*p* = 0.073) cortices. Furthermore, no significant correlations were revealed in the TBI group between LNS delta and MD delta in the CC tracts to the precentral (*p* = 0.970), postcentral (*p* = 0.801), frontal (*p* = 0.811), parietal (*p* = 0.883), temporal (*p* = 0.978), or occipital (*p* = 0.947) cortices. For the control group, no significant associations were revealed between DS delta and MD delta in the CC tracts to the precentral (*p* = 0.637), postcentral (*p* = 0.466), frontal (*p* = 0.618), parietal (*p* = 0.393), temporal (*p* = 0.230), or occipital (*p* = 0.890) cortices.

IHTT predicted cognitive performance over time in pediatric patients with TBI (*n* = 19). Ordinary least squares regression models regressed DS delta performance on IHTT speed at T1 and MD delta in CC tracts associated with functional verbal working memory networks (Chai et al., 2018; Østby et al., 2011). Age, binary sex, years of parent education, and GCS scores at hospital admission were included as covariates. Assumptions of normality, minimal outliers, and homoscedasticity were screened and were within acceptable limits. There was minimal autocorrelation in the residuals as suggested by the Durbin-Watson statistic, which yielded a value of 2.34. A minimal degree of multicollinearity was confirmed with VIF (< 2.50) and tolerance (> 0.40) estimates; correlations between IHTT and MD in the CC tract to the frontal (*r* = 0.252), and parietal (*r* = 0.390) cortices were also not suggestive of multicollinearity. Using G*Power, a post-hoc power analysis for two predictors associated with the criterion with medium to large effect sizes at the standard alpha level computed an achieved power that was within acceptable limits (1 – β = 0.88). MD delta in the CC tract to the frontal cortex (β = –0.397, *p* = 0.045) and average IHTT speeds at T1 (β = –0.680, *p* = 0.001), in addition to the covariates, significantly predicted DS delta scores, *F*(6, 12) = 7.43, *p* = 0.002, *R*^2^ = 0.79 (Table 5). A similar model with MD delta in the CC tract to the parietal cortex (β = −0.364, *p* = 0.049) and IHTT at T1 (β = −0.676, *p* = 0.001) also significantly predicted 79% of the variance in the degree of improvement in DS performance from T1 to T2, *F*(6, 12) = 7.30, *p* = 0.002, *R*^2^ = 0.79.

**TABLE 5:** Linear regression model predicting more or less improvement (percent delta) in DS scores from T1 to T2 with IHTT speeds at T1 and percent delta of MD in CC projection to the frontal cortex with covariates for the TBI group (*n* = 19).

	R	R^2^	R^2^_adjusted_	B [95% CI]	β	t
	0.888	0.788	0.682			
Years of parent education				−0.05 [−0.10, 0.01]	−0.35	−1.92
Age at T1				−0.09 [−0.184, 0.01]	−0.44	−2.04
Sex				−0.32 [−0.76, 0.13]	−0.26	−1.56
GCS (Admission)				0.02 [−0.03, 0.06]	0.12	0.84
IHTT Average at T1				−0.03 [−0.04, −0.01]	−0.68	−4.24**
Δ MD CC to Frontal Cortex				−4.67 [−9.21, −0.13]	−0.40	−2.24*
	*F*(6, 12) = 7.43, *p* = 0.002					

*p < 0.05

**p < 0.01. DS, digit span; T1, post-acute (M = 12.38 weeks, SD = 5.08); T2 = chronic (M = 67.01 weeks, SD = 8.11); IHTT, interhemispheric transfer time; MD, mean diffusivity; CC, corpus callosum; TBI, traumatic brain injury.

**FIGURE 4:**
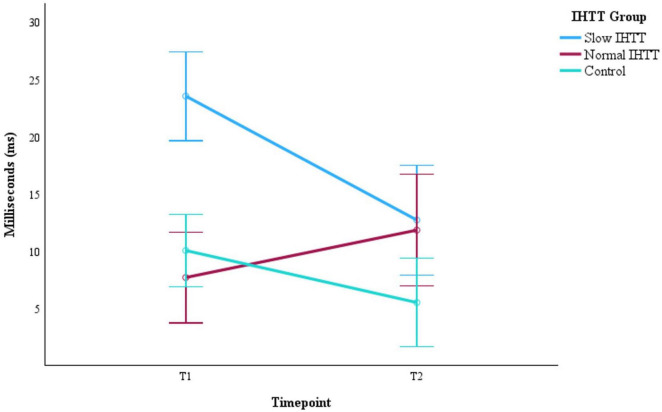
Progression of IHIT speeds from post-acute (T1) to chronic (T2) for the two subgroups of pediatric patients with TBI stratified by IHIT and the typically developing control group with data at both timepoints (*n* = 41). Error bars represent the 95% confidence intervals of the mean. The covariates appearing in the model are evaluated at the following values: Age at T1 = 14.96, Sex = 0.29. IHTT, interhemispheric transfer time; T1 = 8–20 weeks post injury; T2 = 54–96 weeks post injury; TBI, traumatic brain injury.

As a post-hoc analysis on the full sample with IHTT data at both timepoints (*n* = 41), another Group x Time ANCOVA with only age and binary sex at T1 as covariates revealed that IHTT normalized for the Slow IHTT TBI group at T2 (*n* = 12). There was also random attrition for the Normal IHTT TBI (*n* = 11) and control (*n* = 18) groups. Little’s MCAR test confirmed the mechanism of missingness, X^2^ = 1.28, *p* = 0.527; pairwise deletion was deemed appropriate for management of missing data. There was a significant interaction effect, *F*(2, 36) = 9.06, *p* < 0.001, η_*p*_^2^ = 0.355. At T1, the Slow IHTT group was significantly slower than both the control group (*p* < 0.001) and the Normal IHTT group (*p* < 0.001). Over time, the Normal IHTT group (*p* = 0.110) did not exhibit the same improvement in speed as the Slow IHTT (*p* < 0.001) and control (*p* = 0.029) groups (Figure 4). At T2, both TBI subgroups were not different from each other (*p* = 0.795), but the Slow IHTT (*p* = 0.025) and Normal IHTT (*p* = 0.047) groups were both slower than the typically developing control group (Table 6).

**TABLE 6:** Descriptives for IHTT speeds stratified by group over time.

Group	Timepoint	$\bar{\text{x}}$ (*SE*)	Δ$\bar{\text{x}}$ (T1–T2)	Δ$\bar{\text{x}}$ (T1)	Δ$\bar{\text{x}}$ (T2)
Slow IHTT (A)	T1	23.54 (1.91)	10.81***	15.83*** (A–B)	0.88 (A–B)
	T2	12.02 (2.36)			
Normal IHTT (B)	T1	7.71 (1.95)	−4.13	−2.36 (B–C)	6.32** (B–C)
	T2	11.85 (2.41)			
Control (C)	T1	10.07 (1.55)	4.54**	−13.47*** (C–A)	−7.20** (C–A)
	T2	5.53 (1.91)			

**p < 0.05

***p < 0.01; IHTT, interhemispheric transfer time; $\bar{\text{x}}$, mean; SE, standard error; Δ$\bar{\text{x}}$, mean change. T1, post-acute (M = 12.38 weeks, SD = 5.08); T2 = chronic (M = 67.01 weeks, SD = 8.11).

## Discussion

The current longitudinal DTI study in pediatric patients with moderate-to-severe TBI assessed the evolution of mean diffusivity (MD) from the post-acute to the chronic timeframe and evaluated their correlations with long-term neurocognitive outcomes. Indicators of WM organization, both functional and microstructural, were IHTT and MD in CC tracts to frontoparietal regions, which have been proposed as the working memory neural network (Chai et al., 2018; Østby et al., 2011). By evaluating neurocognitive performance and MD trajectories in CC tracts, the current study contributes to the understanding of the natural course of diffuse traumatic injuries and WM repair. Patients were previously stratified into two subgroups by an ERP biomarker (IHTT) measured at the post-acute period following injury and compared to a typically developing control group. Our hypotheses were supported: IHTT stratification identified divergent verbal working memory trajectories that paralleled prior WM microstructural trajectories over time (see Dennis et al., 2017 and Figure 3); change in verbal working memory was correlated with IHTT and changes in WM organization with some regional specificity; and the included biomarkers of interest with covariates explained significant variance (79%) in predicting which children with TBI would experience more or less improvement in verbal working memory at one-to-two-year follow-up (chronic timeframe).

Initial working memory performance within two to five months post-injury was significantly poorer for children with TBI compared to non-injured peers, however patients who exhibited normal (relative to controls) IHTT exhibited notable cognitive recovery over time, comparable with non-injured peers. Longer IHTT at initial measurement was correlated with less improvement in working memory, as well as increased MD in CC tracts to the precentral, postcentral, frontal, and parietal cortices. Improvements were observed only in the pediatric patients who exhibited Normal IHTT at initial measurement, which may reflect the more favorable WM organization relative to the patients with slow IHTT. Increased MD in the evaluated CC tracts from T1 to T2 were strongly associated with slower IHTT speeds at T1 and less improvement in DS performance over time. Normal development of working memory relies on prominent WM tracts like the CC (Li et al., 2022; Tamon et al., 2024) and is strongly associated with reading, mathematics, and academic achievement (Sankalaite et al., 2023). Notably, there was a dissociation between the two working memory tasks of the current study. Converse to the DS working memory task, no significant relationships were revealed between progression of MD in CC tracts and change in the LNS working memory task for the TBI group. Differences in cognitive load may explain the variation, such that sequencing both letters and numbers may rely on interactions with multiple WM tracts as opposed to the task that only required repetition of digits.

The non-injured control group stayed consistent across the presented measurements of working memory but exhibited change in IHTT. There was a significant improvement in speed from initial measurement to follow-up that likely reflects a healthy developmental process, which was also observed for the Slow IHTT TBI subgroup. The Normal IHTT group was not different from the control group in terms of interhemispheric speed at initial measurement but did not exhibit the same significant improvement that their typically developing peers exhibited at follow-up. IHTT normalized for the Slow IHTT group to match the Normal IHTT group, and both TBI subgroups were significantly slower than the control group at follow-up, suggesting that the prognostic utility of this biomarker is time sensitive even though the effects of neurologic trauma may persist into the chronic period.

Interhemispheric brain communication has been proposed to not only transfer necessary information between the brain, but also contribute significantly to the development of integrated lateralized functions (Ravignani et al., 2022). For example, verbal working memory requires the integration of sensory information from both ears and coordination of bilateral motor planning (e.g., subvocalizations) for rehearsal in concert with higher-order cognitive cortices for successful execution. Brincat et al. (2021) studied the association between WM organization and brain wave activity; researchers suggested that the two prefrontal cortices briefly sync beta and theta oscillations to physically transfer traces of visual working memory across hemispheres, which is critical for quickly and successfully engaging in many real-world behaviors (e.g., driving, sports). More efficient interhemispheric communication has been associated with larger callosal diameter and increased density of myelinated fibers for faster conduction (Ravignani et al., 2022). Following TBI, a meta-analytic review of neuroimaging modalities proposed that reduced FA and increased MD in the chronic phase of recovery is suggestive of axonal injury and demyelination (Amyot et al., 2015). There has been evidence of limited endogenous remyelination in the central nervous system, although the reasons for failure are not completely understood. An area of research that focuses on enhancing this process uses molecular techniques, such as RNA interference (RNAi) and monoclonal antibodies that target signaling components of myelin for recovery, although most of this work has not been focused on brain trauma (Huntemer-Silveira et al., 2021). Another promising treatment revealed changes in FA and MD in the precentral cortex and superior longitudinal fasciculus that correlated with verbal working memory improvements in adolescents with moderate-to-severe TBI in response to eight weeks of directed cognitive training (Verhelst et al., 2019). The results of the current study may be helpful in improving rehabilitation effectiveness by identifying the subsets of pediatric patients following brain trauma who may respond most favorably to these types of treatments.

The strengths of the current study include a complex research design (mixed-model factorial). We were able to track changes in WM CC organization and working memory within and between two subgroups of children with brain trauma to study the one-year trajectory of recovery following TBI in a sample engaged in active neurodevelopment. Moreover, the matched control group enabled comparisons of these trajectories to typical development. Another strength of the current study was repeated measurements of interdisciplinary assessments (e.g., MRI, EEG, neurocognition) to comprehensively approach characterizing the relationship between WM disorganization and neurocognition in pediatric moderate-to-severe TBI.

To better understand causality, future longitudinal studies should investigate changes between IHTT, WM organization, and working memory at more frequent assessments longitudinally to characterize how these changes progress. The current study was limited to evaluating linear trends as there were only two points of data collection which limits our capacity to evaluate the extent that symptoms following medically or clinically complicated pediatric TBI may wax and wane, and if there is instability, to what extent would this variability be associated with the indicators of WM disorganization presented above. Additionally, IHTT as a biomarker still needs to be replicated in an independent sample to further support the model proposed above, which measures pathobiology to potentially make outcome predictions at the patient-level (Guerriero and Schlaggar, 2017).

This study is also limited by modest sample size, which emphasized the need to minimize data loss in the management of missingness and restricted the number of outcomes that could be evaluated in conjunction with the current findings. For example, measures of academic performance and parent- or self-endorsements of functional independence may have generated additional insights about how affected endophenotypes may manifest in a child’s life. Future developmental studies should incorporate increased sample sizes from a wider range of backgrounds to appropriately evaluate the potential of additional environmental and psychosocial influences (e.g., census-level indicators of neighborhood health, multidimensional measures of parenting behavior, adverse childhood experiences) to predict neurocognitive outcomes beyond sex, age, and years of parent education. Contemporary models of neurodevelopmental disorders need to consider the heterogeneity of symptoms, cumulative risk factors over time, and the influence of environmental predispositions (Moll, 2024).

Evaluating functional and microstructural indicators of WM organization early in recovery are potential prospective methods to improve the risk-stratification problem following acute TBI. Thus, exploring whether interventions aimed at improving changes in WM microstructure (e.g., cognitive training, neuromodulation) can lead to improvements in working memory may be beneficial, preemptively targeting children with slower IHTT. A recent evidence-based review of 16 randomized controlled trials focused on cognitive rehabilitation interventions post-moderate to severe TBI revealed level 2 evidence for improvements in self-regulation in response to problem-solving therapy involving clear thinking components and level 1b evidence for using mobile devices as compensatory strategies in the Cognitive Applications for Life Management (CALM) program to improve anger, aggressive behaviors, and subjective distress (Flores-Sandoval et al., 2024). Activity-dependent myelination is a dynamic process dictated by experience and the multi-disciplinary fields of pediatric neuropsychology, psychiatry, and neurology may be well-equipped to address the chronic challenges presented by pediatric TBI (Babikian et al., 2015).

Lastly, there may be several other mechanisms that underlie differences observed in white matter disorganization that were not measured directly in the current study. In addition to the axonal shearing forces and significant atrophy of WM tracts after initial injury (Wu et al., 2011), a diverse set of secondary injury processes may also follow for years (Dinkel et al., 2014; Giza and Hovda, 2014). This neurometabolic cascade may be underlying the maintenance of symptoms once the primary injury has resolved through disordered microtubule arrangement, buildup of cellular debris, or demyelination (Treble et al., 2013). Other potential subacute and chronic mechanisms include abnormal proliferation and reactive glial response, or excitotoxic neurochemical changes (e.g., glutamate). Distinctly, acute mechanisms such as blood vessel damage that could lead to reduced blood flow and ischemia or swelling from inflammation to increase intracranial pressure may be interacting in differing degrees to collectively impede a child’s capacity to successfully reintegrate into their lives over time. Promising findings must be translated to clinical practice in primary care settings to aid in identification, then further to specialty providers and community clinical settings to intervene and manage atypical neurodevelopment.

## Data Availability

The raw data supporting the conclusions of this article will be made available by the authors, without undue reservation.
